# Integrin, Exosome and Kidney Disease

**DOI:** 10.3389/fphys.2020.627800

**Published:** 2021-01-25

**Authors:** An-Ran Shen, Xin Zhong, Tao-Tao Tang, Cui Wang, Jing Jing, Bi-Cheng Liu, Lin-Li Lv

**Affiliations:** Institute of Nephrology, Zhongda Hospital, Southeast University School of Medicine, Nanjing, China

**Keywords:** exosome, integrin, extracellular vesicle, kidney disease‐diagnosis, kidney disease‐therapy

## Abstract

Integrins are transmembrane receptors that function as noncovalent heterodimers that mediate cellular adhesion and migration, cell to cell communication, and intracellular signaling activation. In kidney, latency associated peptide-transforming growth factor β (TGF-β) and soluble urokinase plasminogen activator receptor (suPAR) were found as the novel ligands of integrins that contribute to renal interstitial fibrosis and focal segmental glomerular sclerosis glomerulosclerosis (FSGS). Interestingly, recent studies revealed that integrins are the compositional cargo of exosomes. Increasing evidence suggested that exosomal integrin played critical roles in diverse pathophysiologic conditions such as tumor metastasis, neurological disorders, immunology regulation, and other processes. This review will focus on the biology and function of exosomal integrin, emphasizing its potential role in kidney disease as well as its implications in developing novel therapeutic and diagnosis approaches for kidney disease.

## Introduction

Integrins are transmembrane receptors that function as noncovalent heterodimers. There are 24 distinct integrin receptors that can recognize and bind to multiple ligands such as extracellular matrix (ECM) proteins, thereby mediating cell adhesion and intracellular signaling (Moreno-Layseca et al., 2019). Other novel ligands include latency associated peptide-transforming growth factor β (L-TGF-β) and soluble urokinase plasminogen activator receptor (suPAR) were found to bind to integrin and participated in the pathogenesis of kidney disease. Moreover, activated integrins on diverse kidney cells in pathological conditions participated in macrophage and fibroblast activation which played important roles in diverse kidney diseases.

Exosomes are small extracellular vesicles (EVs) secreted by nearly all types of cells which are originally considered to be the garbage bins of cells to excrete unwanted materials (Johnstone et al., 1987). Recently, accumulating studies have demonstrated that exosomes participated in crosstalk between cells and also mediated communications between organs. Moreover, exosomes can serve as vectors of therapeutics and facilitate disease diagnosis in a noninvasive way (Kalluri and LeBleu, 2020). Interestingly, integrins are revealed as the important compositional components of exosomes which take responsibility for those novel functions of exosomes.

The diverse pathophysiological roles of exosomal integrins varied from guiding the homing of exosomes (Hoshino et al., 2015), signal transmission (Kalappurakkal et al., 2019), causing phenotype transition of recipient cells (Lu et al., 2018) to cell adhesion (Genschmer et al., 2019) and migration (Sung et al., 2015). Although studies have revealed essential roles of exosomal integrin in oncology, neurology, and immunology, its role in kidney pathophysiology remains unclear. Thus, exploring the role of exosomal integrin in kidney disease would be helpful in understanding the mechanism of kidney disease and identifying novel diagnosis and treatment strategies. Here, we review the biology and functions of integrin as well as integrin carried by exosomes. Pathophysiologic roles of exosomal integrin in diverse diseases are also discussed, especially the role and potential applications in therapy and diagnosis of kidney diseases.

## Biology and Function of Integrin

### Structure and Endocytic Trafficking of Integrin

Integrins are transmembrane heterodimers which express conservatively in almost all cell types. Integrin family was initially discovered on immune cells and mediates leukocyte extravasation by binding to intercellular cell adhesion molecule-1 (ICAM-1) on vascular endothelial cells (capturing intravenous immune cells) (Dustin, 2019). Integrins display three distinct conformations (bent, extended close, and extended open), while the activity is usually observed in the extended conformation (Campbell et al., 2020). Integrins can be categorized into 24 subtypes formed by 18 types of α subunits and eight types of β subunits. Among them, integrin αv, α6, and β1 are known for pairing with diverse subunits (Moreno-Layseca et al., 2019). Each integrin subunit contains a large extracellular domain, single-time transmembrane domain, and often rather short cytoplasmic domain (Humphries et al., 2006). Studies have shown that certain integrin subtypes are expressed on specific tissue or cell or bind to certain types of cells. For example, integrin β6 is expressed in a few subset of epithelial cells (Breuss et al., 1993). Integrin α6β1 and integrin α6β4 targeted to lung fibroblast while integrin αvβ5 targeted liver Kupffer cell (Hoshino et al., 2015). However, the dominant subtype and the abundance of integrin in a specific cell type could change under certain injury conditions. For example, the dominant integrin subtype in podocyte is α3β1 (Kreidberg et al., 1996), while it changes into αvβ3 under focal segmental glomerulosclerosis (FSGS) situation (Hayek et al., 2017).

Although the expression levels of integrin are quite stable in certain cells and tissues, they are continuously trafficking from cytoplasm to surface membrane by diverse complex pathways including the Rab family of small GTPase (Moreno-Layseca et al., 2019). This process includes integrin endocytosis into early endosome which then traffic to late endosome and recycle to the cell surface, or alternatively transport to multivesicular bodys (MVBs) and subsequent lysosome for degradation (Rainero and Norman, 2013). Generally, majority of endocytic integrins travel back to the cell surface while small fractions target to degradation (De Franceschi et al., 2015). Interestingly, integrin endocytic trafficking process shares a common intracellular structure, MVBs, with exosome (Rainero and Norman, 2013), thus integrins could also be transported *via* exosome which has been demonstrated in recent studies. A study using gene ontology (GO) and Kyoto Encyclopedia of Genes and Genomes (KEGG) pathway analyses also revealed a correlation between integrin signaling and exosome secretion (Zhang et al., 2020).

### Ligands of Integrin

Tremendous efforts have been invested in integrin ligand discovery, the well-known integrin ligands belong to ECM proteins, newly identified ligands include L-TGF-β compound and suPAR (Humphries et al., 2006; Campbell et al., 2020; Hayek et al., 2020). According to the binding motif on the ligand, integrins can be classified into five types, among which, the most common type is RGD-binding integrins which belong to αv integrins. Thus, studies have been using RGD peptide to inhibit integrin αv subtype binding (Hoshino et al., 2015). Although multiple ligands have been discovered, most of which are non-specific ligands that can bind to more than one types of integrins and mediate cell-cell adhesion in integrin A-ECM-integrin B format (Sung et al., 2015). This suggests that in studying of integrin function under certain conditions, not only specific ligands but also the existence of other integrin subtypes should be considered.

### Biological Functions of Integrins

Integrins bare different biological functions according to their diverse localizations throughout the body including cellular adhesion and migration, regulation of cellular phenotypes, cell to cell communication, and intracellular signaling activation (Table 1).

**Table 1:** Expression and function of major integrin subtypes.

Integrin subtype	Expression	Function	Reference
*αvβ3*	podocytes, endothelial, and cancer stem cells	Bind to suPAR, promote FSGS, cancer progression	Hayek et al., 2017; Nieberler et al., 2017
*αvβ6*	epithelial cells and tumor cells	Activate TGF-β1, tumor progression, and metastasis	Breuss et al., 1993; Nieberler et al., 2017
*αvβ8*	kidney glomerular mesangial cells, brain, and placenta	Activate TGF-β1, inhibit cell growth, spreading, and focal contact formation	Cambier et al., 2000; Campbell et al., 2020
*β1*	multiple cell types	Cell adhesion, maintain cell polarity, regulate cell proliferation, and cell cycle	Liu et al., 2018a; Kormann et al., 2020
*α3β1*	kidney tubular epithelial cells, glomerular endothelial cells, and podocytes	Kidney development and cell anchorage	Kreidberg et al., 1996; Glynne et al., 2001
*α4β1*	reticulocytes	Blood vascular related disease progression	Rieu et al., 2000
*α5β1*	endothelial cells and cancer stem cells	Vascular morphogenesis, cancer, and metastasis	Zovein et al., 2010; Nieberler et al., 2017
*α6β1*	ureteric bud	Maintains the structural integrity of the kidney collecting system	Viquez et al., 2017
*α6β4*	cancer cells and epithelial cells	Lung organotropic metastasis	Hoshino et al., 2015

Integrins were first reported as adhesion molecule in the immune system (Springer, 1990), which represented the basic function of integrins. Many studies have revealed that integrins mediated adhesion between cells or cell to ECM. In kidney, tubular epithelial cells bind to each other on the lateral surface through integrins and bind to ECM on the basal surface by integrins as well (Glynne et al., 2001). Integrins and ECM interaction is also important for cells that underwent polarization during differentiation. Studies have showed that integrin β1 connects ECM and cytoskeletal protein on one side of the cell, which then forms the basal membrane of cells during polarization, such as epithelial cells and endothelial cells (Lee and Streuli, 2014; Moreno-Layseca et al., 2019). Besides, dysregulation of integrin or redistribution have a great impact on cellular apical and basal polarization under injury or cancerization (Glynne et al., 2001; Liu et al., 2018a).

Moreover, integrins are associated with certain cellular phenotype and function under pathological conditions. In contrast to associated nephropathy, integrin αvβ6 increased in injured tubular cells, while remains at low baseline level in normal tubules. Interestingly, injured tubular cells with high integrin were able to bind with suPAR which caused further damage (Hayek et al., 2020). Integrins also participate in regulation of cell cycle in numerous pathways as determined by various *in vitro* and *in vivo* studies. Integrin β1, β3, and other subtypes have been well demonstrated to be involved in cellular proliferation (Panchatcharam et al., 2010; Moreno-Layseca and Streuli, 2014; Raven et al., 2017). Integrins mediate the local niche signal which forms spatial checkpoints that enable cells progress into S phase to proliferate. Reversely, some integrin subtypes or isoforms could prevent cells from progressing into cell cycle, thus inhibiting proliferation, such as integrin α6Bβ6 in colon cancer cells (Dydensborg et al., 2009).

Ligand-integrin binding leads to signaling activation intracellularly, including focal adhesion kinase (FAK), RhoA signaling, and Glycosylphosphatidylinositol-anchored proteins (GPI-APs) nanoclustering (Kalappurakkal et al., 2019). These signals can then activate downstream processes. For example, integrin αvβ3 can regulate angiogenesis (Danhier et al., 2012) by promoting HIF-1α expression and subsequent endothelial-mesenchymal transition (EndoMT; Fan et al., 2018). Importantly, recent studies suggested that the immobilization of integrin-binding ligand and integrin conformation was essential to integrin activation (Kalappurakkal et al., 2019; Campbell et al., 2020).

## Integrins as Compositional Cargo of Exosomes

Exosomes belong to small EVs with the size of less than 200 nm. It is excreted into extracellular space and can transfer mRNA, miRNA, lipid, and protein to receptor cells, therefore, mediating crosstalk with neighbor and remote cells (Valadi et al., 2007; Paolicelli et al., 2019; Lv et al., 2020). Exosomes share most of the compositions from the parent cells with certain cargoes selectively sorting into the vesicles. Multiple proteomic studies on exosomes have revealed that proteins that usually present include both membranous protein and luminal protein (van Niel et al., 2018). Notably, integrins are the commonly identified exosome related proteins sorted from the parent cell. Integrin can be transported by exosomes in tumor (Quaglia et al., 2020), the central nervous system (Zhang et al., 2020) or the immune system (Genschmer et al., 2019). As the common compositional cargo of exosomes, integrins were identified as one of the critical functional cargoes of exosomes in different pathophysiological conditions.

## Pathophysiologic Roles of Integrins Transported by Exosomes

**Figure 1:**
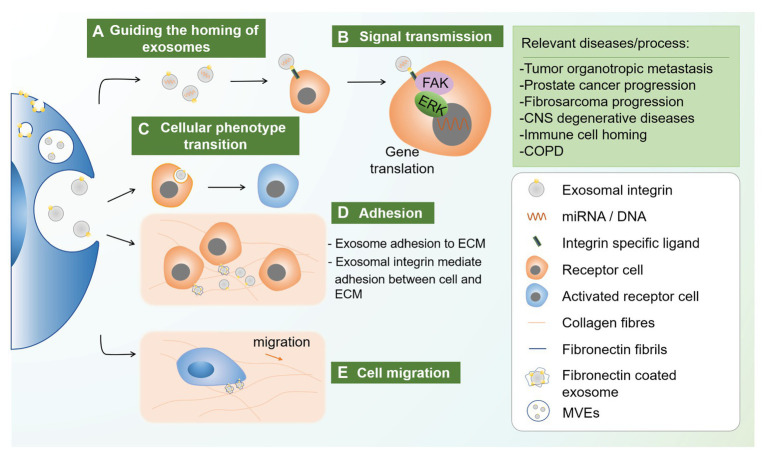
Novel functions of exosomal integrin. Exosomal integrin played diverse roles in different disease conditions including guiding the homing of exosomes, signal transmission, cellular phenotype transition, and cellular adhesion and migration. (a) Exosome cargoes such as miRNA and protein can be delivered to neigbor and distant cells, while specific type of exosomal integrin can guide the vesicles to specific cells through integrin-ligand recognition. (b) Integrin-ligand interatction could activate intracellular signals, for example FAK and ERK-1/2, which initiate relevant gene translation. (c) Apart from intracellular signaling, endocytosis of specific exosomal integrin could also cause cellular phenotype transition of the receipient cells. (d) Exosomal integrin mediated exosome adhesion to extracellular matrix (ECM) through integrin-ECM binding. (e) Moreover, ECM, for example, fibronectin, could be coated on exosome through a process involving endocytosis of integrin αvβ1-fibronectin complex which then sorted into MVEs. FN-coated exosomes secreted and bind to collagen fibrils, which can then coupled to cellular integrin receptors. This adhesion ensures the stable adhesion to ECM during migration. CNS, central nervous system; COPD, chronic obstructive pulmonary diseases; MVEs, multivesicular endosomes.

Exosomal integrin is a versatile form that functions actively in different pathophysiological conditions (Table 2) which attributed to guiding the homing of exosomes, signal transmission, phenotype transition of the recipient cells, and cell adhesion and migration (Figure 1). Here, the roles of exosomal integrins in tumor, neurological disorders, immunology, and other diseases were discussed.

**Table 2:** Diverse functions of exosomal integrin.

Diseases/process	Pathophysiologic role of exosomal integrin	Reference
**Oncology**		
Tumor metastasis	Organotropic metastasis and tumor microenvironment formation	Hoshino et al., 2015
Prostate cancer	Cell migration and induce integrin expression through uptake of exosomes	Fedele et al., 2015
Prostate cancer	Macrophage polarization and transferring αvβ6 integrin from cancer cells to monocytes through exosomes	Lu et al., 2018
Prostate cancer	Determined cargo loading of exosomes which promoted cancer cell formation	Quaglia et al., 2020
Fibrosarcoma	Promoted cell migration	Sung et al., 2015
**Neurology**		
Demyelination	Oligodendrocyte precursor cells proliferation	Zhang et al., 2020
CNS diseases	Therapeutic protein delivery, exosome uptake, and spread of viral proteins to the brain	Yuan et al., 2017
**Pulmonary**		
COPD	Mediated exosome adhesion to extracellular matrix	Genschmer et al., 2019
**Gut**		
lymphocyte homing	Exosomal integrin α4β7 target high endothelial venule (HEV) endothelial cells causing diminish in lymphocyte homing niche	Myint et al., 2020

### Tumor Formation, Progression and Metastasis

In recent years, the role of exosomes in mediating tumor formation and progression has been well studied (Li et al., 2019), integrins are among the important cargoes contributing to the process. It is reported that integrin αvβ3 containing small EVs from prostate cancer cells was transferred to recipient cancer cells which induced aggressive phenotype changes (Quaglia et al., 2020). Cancer cell migration was proved to be mediated by autocrine secretion of exosomes. Fibrosarcoma cells-derived exosomal integrins and fibronectin forms adhesion assembly that mediated adhesion between cell and ECM, therefore, promoted cell motility with specific directions (Sung et al., 2015). Fibronectin was coated on exosome through binding with exosomal integrin, which then anchor to cell membrane on one side and ECM on the other and contributed to cellular adhesion (Sung et al., 2015). Moreover, it is demonstrated that exosomes transfer integrin αvβ6 from cancer cells to monocytes, which promoted M2 monocyte polarization and prostate cancer progression (Lu et al., 2018).

Besides, integrins on tumor-derived exosomes can determine organotropic metastasis by forming tumor microenvironment (TME) in specific organ tissues as they uptake the exosomes (Hoshino et al., 2015). They demonstrated the specific integrin subtype directed organotropic metastasis for the first time, such as integrins α6β4 and α6β1 mediated lung metastasis, while integrin αvβ5 mediated liver metastasis (Hoshino et al., 2015). The targeting properties of exosomal integrin were attributed to the activation of the Src-S100A4 axis (cancer associated genes) by exosomal ITGα6β4 in lung fibroblasts during pre-metastatic niche formation (Hoshino et al., 2015). These findings indicated the great therapeutic potential by targeting certain integrin subtype that was involved in tumor metastasis.

### Neurological Disorders

Exosomal integrins participate in the development of neurological disorders and are active in multiple trans-cellular communication processes. Proteomic analysis proved that integrin αvβ3 was upregulated in interleukin-1β (IL-1β) stimulated astrocyte-derived EVs (ADEV) and significantly increased uptake of ADEV in neurons, while integrin αvβ3 blocking partially suppressed this process (You et al., 2020). Exosomal integrins also contributed to the recovery of central nervous system (CNS) degenerative diseases, which was associated with the proliferation of oligodendrocyte precursor cells (OPCs; Zhang et al., 2020). Moreover, in therapeutic studies, macrophage derived exosome contained the integrin subtype lymphocyte function-associated antigen 1 (LFA-1). This facilitated macrophage derived exosome to overcome the blood-brain barrier and deliver therapeutic protein cargos specifically to treat CNS diseases (Yuan et al., 2017).

### Immunology Regulation and Others

Integrin was first discovered as adhesion molecules in immune cells that mediated extravasation (Springer, 1994). Similar to forming TMV for tumor metastasis, exosomal integrins were also involved in immune cell homing (Myint et al., 2020). Study showed that integrin α4β7 on T cell-derived exosomes guided the exosomes homing to the intestine through binding to mucosal addressin cell adhesion molecule-1 (MAdCAM-1; Mora et al., 2003). On the other hand, integrin α4β7-expressing T cell exosomes could suppress MAdCAM-1 expression which, therefore, inhibited subsequent lymphocyte homing to the gut (Park et al., 2019).

Besides, activated polymorphonuclear leukocyte (PMN) derived exosomes were capable of targeting ECM through MAC-1 (αMβ2 integrin). This caused activation of neutrophil elastase (NE) that was coated on exosomes and lead to ECM degradation (Genschmer et al., 2019). Moreover, during reticulocyte maturation, integrin α4β1, that expressed commonly on the surface of reticulocyte, was cleared from the reticulocyte through exosome secretion. This reduced the risk of blood circulation complications, such as sickle-cell anemia, caused by integrin α4β1 on reticulocytes (Rieu et al., 2000).

## Integrins in Kidney Diseases

### Integrins Expression in Renal Cells

Studies have revealed that integrins are expressed on various types of cells in the kidney including tubular epithelial cell (TECs) (Zhu et al., 2020), fibroblast (Bon et al., 2019), and podocyte (Hayek et al., 2017).

Tubular epithelial cells are the primary cellular component of kidney which is susceptible to diverse injuries (Liu et al., 2018b). TECs express αv and β1 integrins under normal conditions (Bon et al., 2019), while integrin αv, β1, and β6 are the dominant subtypes with injury (Hayek et al., 2020). *ITGβ6* (gene of integrin β6) was rarely identified in normal TECs but rapidly increased in the format of αvβ6 subtype under injury. Moreover, a study of clinic kidney biopsy concluded that integrin β6 was elevated in the distal tubules in diverse diseased and transplanted kidney (Trevillian et al., 2004). Highly expressed β1 integrins are known to be involved in epithelial cell polarization which traffic from basal membrane to apical membrane under injury (Glynne et al., 2001). This could result in detachment of TECs from basal ECM and impairment of polarization, which caused further injury of tubules and dysregulation of cell secretion, since integrins played a key role in delivering molecules to the right subcellular compartments (Moreno-Layseca et al., 2019).

Fibroblasts are one of the main cellular components in renal interstitial fibrosis, they can migrate to damaged site, transform into myofibroblasts, and produce ECM. Fibroblasts normally express integrin α1, α4, α5, and β1 and turn into integrin α5, β1, and αv under fibrosis situations (Norman and Fine, 1999), among which integrin αv was the dominant type (Bon et al., 2019). Integrin α5 facilitates fibroblasts migration through binding to ECM (Lobert et al., 2010). Interestingly, integrin αv expressed by fibroblasts binds to latent-TGF-β and stimulates subsequent tissue fibrosis (Henderson et al., 2013). This relation between integrin and fibroblasts also presents in other organs such as colon (Peng et al., 2018), skin (van Caam et al., 2020), lung, liver (Reed et al., 2015), and pancreatic duct (Cavaco et al., 2019).

Podocytes are special for their foot processes and integrin α3 plays a critical role in its maturation. *In vivo* study showed that the mutation of murine integrin α3 gene caused abnormal kidney and lung development (Kreidberg et al., 1996). Studies reported that activated β3 integrin on podocytes could initiate FSGS pathology in a suPAR-APOL1-integrin αvβ3 tripartite complex dependent manner. The underlie mechanisms included autophagosomes formation, actin cytoskeleton dysregulation, and cell detachment (Wei et al., 2011).

### Novel Roles of Integrins in Kidney Disease

Recent studies have showed that integrins bind with novel molecules and drive subsequent signaling pathways, including TGF-β and suPAR. Distinct integrins bind with latent-TGF-β which activates TGF-β and downstream signals, such as Smad2/3. These signals can promote interstitial fibrosis in chronic kidney disease (CKD; Meng et al., 2016) and suppress TEC proliferation after injury in acute kidney injury (AKI; Yang et al., 2019). It was demonstrated that the increased TGF-β signaling was initiated in the early stage of AKI which continuously expressed during recovery stage. TGF-β expression in the tubules was companied by integrin β6 and lead to subsequent interstitial fibrosis (Geng et al., 2012). In this regard, integrins may play a prominent role in AKI to CKD transition by activation of TGF-β.

Unlike ECM or TGF-β, suPAR is not stabilized, it is the released version of the podocyte urokinase receptor (uPAR), which function as the cellular receptor for urokinase. suPAR exists in the circulatory system and its increased concentration is associated with acute (Hayek et al., 2020) and chronic kidney injuries (Hayek et al., 2017). Several studies have reported that suPAR primarily binds with β3 integrin on the surface of podocytes (Wei et al., 2011) by way of a tripartite complex of suPAR-APOL1 risk variants-integrin β3 (Hayek et al., 2017). Meanwhile, suPAR bind to TECs through integrin β6 under injured conditions and activated Rac1, which bound to SRp40 at the 5′ end of exon 7 in versican pre-mRNA. Versican then resulted in subsequent fibroblast activation and promoted interstitial fibrosis by activating the CD44/Smad3 pathway (Han et al., 2019). Moreover, suPAR could bind to integrin β1 and β2 which promoted inflammation and tumor progression (Simon et al., 2000).

### Potential Function of Exosomal Integrins in Kidney Disease

Studies from our group and others have demonstrated that TEC released exosomes mediated cross-talk with fibroblasts (Guan et al., 2020) and macrophages (Lv et al., 2020) which contributed to renal inflammation and fibrosis. However, the traveling direction of TEC exosomes to specific cells remains largely unknown. Since integrins are the common compositional cargoes of exosomes, it is reasonable to speculate that integrin may be critical for directing the fate of the exosomes. Indeed, our study showed that integrin αLβ2 (LFA-1) and α4β1 (VAL-4) on exosomes enabled them to adhere to the inflamed kidney (Tang et al., 2019). Thus, integrin on exosomes may be critical for guiding the traveling of TECs exosomes and mediated the cross-talk with specific recipient cells. Besides, due to the critical role of integrins such as integrin αvβ6 and β1 on TECs, integrin carried by exosomes may play an important role in interstitial inflammation and fibrosis. Moreover, podocytes express integrin β3 that binds with soluble particle suPAR (Wei et al., 2011), thus, podocyte may secrete exosomes with β3 integrins and meditate cellular communication in kidney disease.

Since integrin intracellular trafficking shares multiple pathways with exosome packing and releasing, integrin may also be involve in exosome generation (Rainero and Norman, 2013). Knockdown of integrin β4 decreased the concentration of exosomes in the cultured OPCs supernatant and the capacity to proliferate, while supplement of exosomes reversed this capacity (Zhang et al., 2020). This strongly indicated the critical role of integrin in exosome generation and function. Therefore, the role of integrins in exosomes release and cargo loading for kidney cells under pathologic conditions deserve further investigation.

Hence, as the compositional cargo, integrins carried by exosome may mediate specific cell-crosstalk which participate in the pathophysiological process of the kidney.

## Integrins Carried by Exosomes in the Target Therapy and Diagnosis of Disease

Currently, exosome has been demonstrated as the promising engineered nanocarriers in therapy of disease due to its low immunogenicity, biological barrier permeability, and intrinsic targeting properties (Tang et al., 2020). Studies have showed that exosomal integrins may contribute to the properties of targeting delivery of exosomes. Integrins naturally expressed on exosomes could be used to realize target exosome therapy. Recently, it was found that macrophage-derived EV migrated toward inflamed endothelial cells which was mediated by integrin αLβ2 and integrin α4β1 on EVs in kidney (Tang et al., 2019) and brain (Yuan et al., 2017), respectively. Proteomic analysis of macrophage-derived micro vesicle (MV) carried with dexamethasone revealed that integrin αLβ2 (LFA-1) and α4β1 (VAL-4) express distinctly on the surface, which could efficiently direct MV to the inflamed kidney through recognizing ICAM-1 and vascular cell adhesion molecule-1 (VCAM-1) (Tang et al., 2019).

Since exosomal integrins are important for tumor metastasis, it might hold promise in targeted drug delivery for tumor (Qiao et al., 2020). It is demonstrated that non-small cell lung cancer cells could specifically uptake breast cancer (MDA-MB-231) cell-derived exosomes (231-Exo), which was loaded with mRNA-126 that successfully inhibited lung metastasis *in vivo* (Nie et al., 2020). This organotropic process was depended on integrin β4-exosome that specifically targeted surfactant protein C (SPC) on cancer cells. Moreover, EVs can be engineered to express integrin for target therapy. For example, a study used click chemistry method to conjugate integrin αvβ3-specific cRGD peptides to the surface of exosomes. The results showed the engineered exosome efficiently targeted to injured areas in the brain (Tian et al., 2018).

Based on the discovery of disease-specific integrin by various exosome proteomic studies, detection of different types of integrin carried by exosomes could be novel biomarkers of diseases. It was found that integrin is among the top 100 protein in urinary EVs proteomic studies including AKI, FSGS, autosomal dominant polycystic kidney disease (ADPKD), etc. (Merchant et al., 2017). According to proteomic analysis, integrin on urinary exosomes showed strong correlation with kidney diseases. For example, integrin signaling was identified as the most canonical represented signaling pathways correlated with inherited glomerular diseases by way of ingenuity pathway analysis (Hogan et al., 2014).

## Conclusions and Perspectives

Exosomal integrin played diverse roles in different disease conditions *via* mediating intercellular crosstalk. Integrins are essential for normal cellular adhesion and polarization, while specific pathogenic subtypes of integrins have the potential to trigger renal inflammation and fibrosis *via* activating TGF-β, epithelial-mesenchymal transition (EMT) signaling, FAK and mitogen-activated protein kinases (MAPKs). However, the role of exosomal integrin in kidney disease remains largely unknown. Exosomal integrin may contribute to the injury and repair processes of kidney disease as the novel format of integrin *via* mediating cellular communication and downstream signaling activation. In addition, integrins may also hold the potential to participate in intracellular exosome secretion and cargo loading which may provide a promising approach for engineering of exosome for diagnosis and therapeutic purpose.

The guiding effect of specific exosomal integrin was demonstrated in tumor or immune cells. Despite that integrin can direct the destination of exosomes, the underlie mechanism require further investigation. Nevertheless, the guiding effect of exosomal integrin provided an important pathway for developing target therapy for kidney diseases. Further investigation in the role of diverse exosomal integrin subtypes in cellular communication may allow the construction of specific targeting exosome for precise treatment of kidney disease.

## Author Contributions

A-RS wrote the manuscript. L-LL conceived the concept and contributed to the writing of the manuscript. All authors contributed to the literature review and approved the submitted version.

### Conflict of Interest

The authors declare that the research was conducted in the absence of any commercial or financial relationships that could be construed as a potential conflict of interest.
